# Re-Evaluation and Retrospective Comparison of Serum Neutralization Induced by Three Different Types of Inactivated SARS-CoV-2 Vaccines

**DOI:** 10.3390/vaccines12111204

**Published:** 2024-10-24

**Authors:** Weiyu Jiang, Jianbo Wu, Jiaying He, Anqi Xia, Wei Wu, Yidan Gao, Qianqian Zhang, Xiaofang Peng, Qiaochu Jiang, Song Xue, Qiao Wang

**Affiliations:** 1 Key Laboratory of Medical Molecular Virology (MOE/NHC/CAMS), Shanghai Institute of Infectious Disease and Biosecurity, Shanghai Frontiers Science Center of Pathogenic Microbes and Infection, School of Basic Medical Sciences, Shanghai Medical College, Fudan University, Shanghai 200032, China; 21111010061@m.fudan.edu.cn (W.J.); 21111010079@m.fudan.edu.cn (A.X.); 13061915085@163.com (W.W.); 20211010051@fudan.edu.cn (Y.G.); 22211020016@m.fudan.edu.cn (X.P.); 17301010038@fudan.edu.cn (S.X.); 2 Department of Laboratory Medicine, Huashan Hospital, Shanghai Medical College, Fudan University, Shanghai 200040, China; 3 Microbiological Testing Department, Baoshan District Center for Disease Control and Prevention, Shanghai 201901, China; 21211010054@m.fudan.edu.cn; 4 Department of Clinical Laboratory, Shandong Cancer Hospital and Institute, Jinan 250117, China; qdzqqian@163.com; 5 Department of Dermatology, Huashan Hospital, Fudan University, Shanghai 200040, China; 18301050216@fudan.edu.cn

**Keywords:** SARS-CoV-2, inactivated SARS-CoV-2 vaccines, variants of concerns (VOCs), serum neutralization, newly emerging variants

## Abstract

Background: During the COVID-19 pandemic, three different types of inactivated SARS-CoV-2 vaccines, namely BBIBP-CorV, WIBP-CorV and CoronaVac, were manufactured and used for vaccination in China. However, as far as we know, no comparison of their induced serum neutralization has been carried out so far, possibly due to the regional difference in vaccine distribution, the difficulty in undertaking a comprehensive evaluation, and the intention to avoid unnecessary bias in populations for a certain type of inactivated vaccine.Methods: Since all three of these inactivated vaccines are no longer produced and used for vaccination, here, we retrospectively compared the serum neutralizing activities induced by these three different types of inactivated SARS-CoV-2 vaccines. Results: Compared with unvaccinated uninfected control donors, primary inactivated vaccination (232 donors) induced increased serum neutralizing titers against wildtype SARS-CoV-2 in around 70% of donors during the first 100 days. However, the neutralization effect waned quickly after 100 days, and significantly diminished against Delta and Omicron (B.1.1.529) variants. Moreover, the newly emerged Omicron variants, such as BA.2.75, BA.4/5, BF.7, BQ.1.1, and XBB, almost fully evaded the induced serum neutralization activity. Conclusions: These three distinct types of inactivated vaccines, namely BBIBP-CorV, WIBP-CorV, and CoronaVac, induced serum neutralization in most vaccinated populations but in a short-term and variant-evaded manner with no significant difference among these inactivated vaccines.

## 1. Introduction

The coronavirus disease 2019 (COVID-19) pandemic has caused approximately 800 million infections and around 7 million deaths worldwide. Immediately after the severe acute respiratory syndrome coronavirus 2 (SARS-CoV-2) emerged in the city of Wuhan, China, in December 2019, the global effort to develop vaccines against COVID-19 was immediately initiated. In the US and Europe, the novel mRNA vaccine strategy was established, and the mRNA-based vaccines, BNT162b2 developed by Pfizer Inc. (New York, NY, USA) [1] and mRNA-1273 by Moderna (Cambridge, MA, USA) [2], were widely used. However, in China, a traditional vaccine strategy was adopted and three types of inactivated-pathogen vaccines were developed, including BBIBP-CorV developed by Sinopharm (Beijing Bio-Institute of Biological Products, Beijing, China) [3], WIBP-CorV developed by Sinopharm (Wuhan Institute of Biological Products, Wuhan, China) [4], and CoronaVac developed by Sinovac Biotech Ltd. (Beijing, China) [5]. The production of all three of these inactivated vaccines has ceased and they are currently not in use any more.

Both mRNA vaccines and inactivated vaccines were available or approved around the same time. The U.S. Food and Drug Administration (FDA) issued an emergency use authorization (EUA) for the two mRNA vaccines, Pfizer–BioNTech’s BNT162b2 and Moderna’s mRNA-1273, on 11 December 2020 and 18 December 2020, respectively [6,7]; meanwhile, the three inactivated vaccines, BBIBP-CorV, WIBP-CorV, and Sinovac’s CoronaVac, were approved for general-population vaccination by China’s National Medical Products Administration (NMPA) on 31 December 2020, 5 February 2021, and 25 February 2021, respectively [8,9,10].

For the BBIBP-CorV vaccine, the SARS-CoV-2 HB02 strain was used and each 0.5 mL dose was composed of 6.5 U (4 μg) of inactivated SARS-CoV-2 antigens (inactivated with 1:4000 vol/vol β-propiolactone) and 0.4 mg aluminum hydroxide adjuvant in phosphate-buffered saline (PBS) [11]. Originally, three SARS-CoV-2 strains, namely 19nCoV-CDC-Tan-HB02 (HB02), 19nCoV-CDC-Tan-Strain03 (CQ01), and 19nCoV-CDC-Tan-Strain04 (QD01), were isolated from the bronchoalveolar-lavage samples or throat swabs of hospitalized patients during the COVID-19 outbreak in Wuhan, while, among these three viral strains, only the HB02 strain showed optimal replication and generated the highest virus yields in Vero cells [11].

For the WIBP-CorV vaccine, the viral strain SARS-CoV-2, WIV04, isolated from a patient in the Jinyintan Hospital in Wuhan and cultivated in a Vero cell line for propagation, was used. Each dose (0.5 mL per dose) was prepared by mixing 5 μg of viral antigen (inactivated with 1:4000 vol/vol β-propiolactone) and 0.5 mg aluminum hydroxide adjuvant [12].

For the CoronaVac vaccine, each dose (0.5 mL) contained 3 µg of inactivated SARS-CoV-2 antigens (CZ02 strain) (inactivated with β-propiolactone and complemented by formaldehyde) and 0.225 mg aluminum hydroxide in PBS [13,14,15].

Theoretically, for inactivated vaccines, the inactivated dead form of the pathogens ensures a better safety profile for vaccination with reduced adverse effects. However, the inactivation process itself, such as with formalin or β-propiolactone treatment to inactivate viruses, might reduce the antigen immunogenicity, leading to lower efficacy of vaccination [16,17]. Consistent with this theory, several population-based observational studies showed that, although the inactivated SARS-CoV-2 vaccines provided substantial protection against “severe COVID-19”, the inactivated SARS-CoV-2 vaccines, compared with the mRNA vaccines, induced lower levels of serological antibodies, with lower vaccine effectiveness and a lower level of protection [18,19,20,21,22].

Although a comparison has been performed between the inactivated vaccines (mainly CoronaVac) and mRNA vaccines, there is almost no comparison among these three inactivated vaccines manufactured and widely administered in China. The reasons for this are probably as follows: (1) The supply and distribution of the three inactivated vaccines show regional differences. Especially at the beginning of the pandemic, due to the great demand for SARS-CoV-2 vaccines, the limited vaccines were uniformly distributed. Therefore, in some cities, only one type of inactivated vaccine was available for vaccination during a certain period of time. Consequently, it was not easy to recruit volunteers for all three types of inactivated vaccines. (2) Comparison might inspire public discussion on the advantages and disadvantages of a certain type of vaccine, generating strong but unnecessary preferences or biases. (3) Evaluation of vaccine efficacy is a comprehensive and systematic piece of work, which includes serum antibody titers, serum neutralizing activity, cellular adaptive responses, side effects after vaccination, protection against severe disease and death, and so on. Partial evaluation might lead to an incorrect conclusion, making the public hesitate to get vaccinated. For example, the decrease in serum antibody titer does not mean that the vaccine has no protective effect, which might be provided by memory B cells and T cells [23,24,25].

Nowadays, all three of these inactivated vaccines based on the ancestral SARS-CoV-2 (Wuhan-Hu-1 strain) have ceased production and are currently not in use any more, and booster vaccines against Omicron and its variants, in China, have mainly been switched to recombinant protein vaccines and mRNA vaccines. Therefore, our re-evaluation and retrospective comparison of these three inactivated vaccines would not generate any negative social effect or any unnecessary bias.

Here, we used 239 serum samples collected from August to September 2021 (87 individuals for the BBIBP-CorV vaccine, 68 individuals for the WIBP-CorV vaccine, 77 individuals for the CoronaVac vaccine, and 7 uninfected unvaccinated individuals), and focused on analyzing their serum neutralizing activity by using in vitro pseudovirus-based neutralization assays. In general, similar levels of serum neutralization were observed for these three different types of inactivated vaccines. Importantly, the serum neutralizing activity declined rapidly after three months post-vaccination. Compared with the wildtype SARS-CoV-2, the serum neutralizing activity against the Omicron (B.1.1.529) variant, but not Delta, was significantly decreased. Moreover, some emerging Omicron variants, such as BA.2.75, BA.4/5, BF.7, BQ.1.1, and XBB, almost fully evaded the serum neutralization induced by the primary inactivated vaccination.

## 2. Methods

### 2.1. Blood Sample Collection

**Figure 1:**
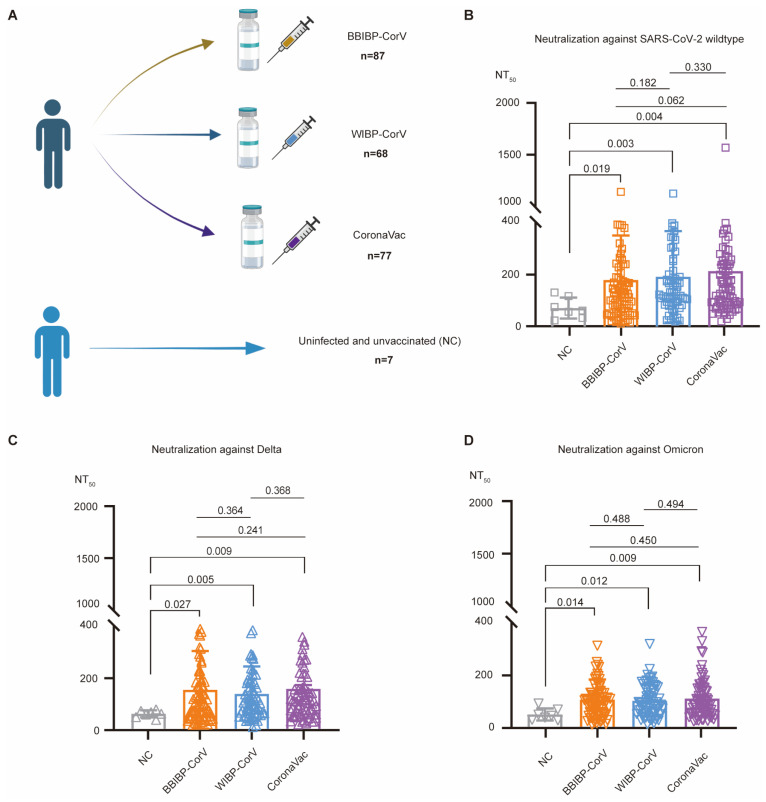
Three distinct types of inactivated vaccines elicited significantly higher levels of serum neutralization against wildtype SARS-CoV-2, Delta, and Omicron (B.1.1.529). (**A**) Recruitment of donors. In total, 239 volunteers were selected for this study, including 87 BBIBP-CorV vaccinees, 68 WIBP-CorV vaccinees, 77 CoronaVac vaccinees, and 7 uninfected, unvaccinated, negative-control (NC) donors. (**B**–**D**) Comparison of serum neutralizing titers against wildtype SARS-CoV-2 (square, **B**), Delta (up-pointing triangle, **C**), and Omicron (B.1.1.529) (down-pointing triangle, **D**). In each graph, each dot represents a donor, with his/her serum neutralizing titer (NT_50_ values on the *y*-axis) calculated based on in vitro neutralization assays. Donors receiving distinct types of inactivated vaccines are shown on the *x*-axis and represented by different colors: gray, uninfected unvaccinated donors as negative controls (NC); orange, BBIBP-CorV-vaccinated donors; blue, WIBP-CorV-vaccinated donors; and purple, CoronaVac-vaccinated donors. The serum neutralizing activity (NT_50_) of these four groups of donors were compared with each other using Kruskal–Wallis test, with the calculated *p*-values shown above the column.

The volunteer recruitment and blood draws were performed at the Shanghai Medical College of Fudan University under a protocol approved by the Fudan University School of Basic Medical Sciences Ethics Committee (FUSBMSEC) (approval number: 2020-007). All volunteers provided written consent for blood collection and the subsequent experiments. All blood samples were collected from 253 volunteers during August 20 to September 2, 2021. Among these donors, 14 individuals received only one dose of the vaccine or two doses of mixed inactivated vaccines and were excluded from this study. Among the selected 239 volunteers, 232 individuals finished the primary vaccination (two doses of vaccination), including 87 individuals who received two doses of BBIBP-CorV, 68 individuals with two doses of WIBP-CorV, and 77 individuals with two doses of CoronaVac (Figure 1A). The production of all three of these inactivated vaccines has ceased and they are currently not in use any more. Seven donors did not receive any vaccination against SARS-CoV-2, and their serum samples were used as negative controls (NC) (Figure 1A). All donors in this study had no SARS-CoV-2 infection before blood donation due to the strict lockdown policy in mainland China. Blood was collected into a tube without anticoagulant, and after centrifugation of coagulated whole blood, the serum samples were collected, aliquoted, and stored at −80 °C. Before use, sera were heat-inactivated at 56 °C for 30 min.

### 2.2. Generation of Pseudotyped Viruses

The pseudotyped viruses of SARS-CoV-2 and its related variants were generated as described previously [26]. Briefly, the backbone plasmid of pNL4-3.Luc.RE and the plasmid of pcDNA3.1-SARS-CoV-2-S to express the viral spike (S) protein were co-transfected into HEK-293T cells (obtained from Dr. Lu Lu lab, Shanghai, China. [27]) using the VigoFect transfection reagent (Vigorous Biotechnology, Haidian, China). Fresh Dulbecco’s Modified Eagle’s Medium (DMEM, Thermo Fisher Scientific, Waltham, MA, USA) as a complete medium containing 10% heat-inactivated fetal bovine serum (FBS, Thermo Fisher Scientific, USA) and 1% penicillin–streptomycin solution (Thermo Fisher Scientific, USA) was used for cell culture. Before transfection and after transfection, the supernatant was replaced with fresh DMEM (complete medium). After culture for a, further 48 h, the cell supernatant containing pseudoviruses were collected, aliquoted and stored at −80 °C.

### 2.3. Pseudovirus-Neutralization Assay

In vitro neutralization assays using pseudoviruses were performed as previously described [26]. Briefly, Huh-7 cells (obtained from Dr. Lu Lu lab, Shanghai, China. [27]) were seeded by using fresh complete DMEM in a 96-well plate. Serum samples were serially diluted in a 1:2 ratio for eight dilutions in total using pseudovirus soup, with the maximum concentration of 1:40 for all sera. After incubation for 30 min at 37 °C, the mixture was added into the Huh-7 cells. After incubation for 24 h, the cell supernatant was replaced by fresh complete DMEM. After cell culture for a further 36 h, the cell supernatant was removed, while the cells were lysed and subjected to luciferase activity measurement using a Firefly Luciferase Assay Kit (Promega, Madison, WI, USA) according to the manufacturer’s instructions.

### 2.4. Statistical Analyses

The details of the statistical analyses are shown in the figure legends. Based on the pseudovirus-based in vitro neutralization assay results, the 50% neutralization titers (NT_50_) for each serum sample against the SARS-CoV-2 prototype, the Delta variant, and the Omicron (B.1.1.529) variant were calculated using nonlinear regression analysis. In order to assess the statistical significance for comparisons, the Mann–Whitney test or Kruskal–Wallis test was performed by using PRISM software (GraphPad 9.5.0).

## 3. Results and Discussion

### 3.1. Serum Neutralizing Activity Induced by Primary Inactivated Vaccination

Three types of inactivated SARS-CoV-2 vaccines, including BBIBP-CorV [3], WIBP-CorV [4], and CoronaVac [5], were widely used during the COVID-19 pandemic in China (Figure 1A). Although there is a regional difference for the supply and distribution of these vaccines across most parts of mainland China, all three of these types of inactivated vaccines were available for vaccination in Shanghai [28]. According to the Shanghai Municipal Health Commission, the number of people who completed the primary series vaccination reached 23 million, covering 92% of the resident population.

To evaluate and compare the serum neutralizing activity induced by these three inactivated vaccines, we recruited volunteers from the Shanghai Medical College of Fudan University in Shanghai and collected their serum samples. In total, 239 volunteers were selected for this study, including 87 BBIBP-CorV vaccinees, 68 WIBP-CorV vaccinees, 77 CoronaVac vaccinees, and 7 uninfected, unvaccinated, negative-control (NC) individuals (Figure 1A). During that period of time, a series of strict lockdown and infection-tracking measures, referred to as the “zero-COVID” policy, were implemented in China to contain the spread of the SARS-CoV-2 virus within communities. Therefore, all volunteers recruited in this study had no history of SARS-CoV-2 infection at the time of blood donation.

Serum neutralizing activity against wildtype SARS-CoV-2 was first determined using in vitro pseudovirus-neutralization assays, and the 50% pseudovirus-neutralization titer (NT_50_) was calculated for each serum sample. Compared with negative controls (average NT_50_: 70.62 ± 13.49), all three types of inactivated vaccines induced approximately three-fold higher levels of serum neutralizing activity, with their average NT_50_ values being 177.4 ± 18.28 for BBIBP-CorV (*p* = 0.019), 189.9 ± 21.13 for WIBP-CorV (*p* = 0.003), and 211.7 ± 24.26 for CoronaVac (*p* = 0.004), respectively (Figure 1B). However, when we further compared the NT_50_ values among three groups of vaccinees receiving three distinct types of inactivated vaccines, we found no statistically significant difference in the serum neutralizing activity induced by BBIBP-CorV, WIBP-CorV, and CoronaVac (*p* = 0.450~0.494) (Figure 1B). Therefore, we concluded that, based on our recruited vaccinated donors, BBIBP-CorV, WIBP-CorV, and CoronaVac induced similar levels of serum neutralization against wildtype SARS-CoV-2.

### 3.2. Serum Neutralizing Activity Against the Delta and Omicron (B.1.1.529) Variants

We then performed in vitro neutralization assays against pseudoviruses of both the SARS-CoV-2 Delta and Omicron (B.1.1.529) variants.

For the Delta pseudovirus, the average NT_50_ values were calculated as 150.70 ± 16.04 for BBIBP-CorV, 135.20 ± 12.71 for WIBP-CorV, 154.20 ± 15.54 for CoronaVac, and 43.62 ± 9.42 for the uninfected unvaccinated serum samples. Three types of inactivated vaccines induced statistically significantly higher levels (around three-fold, *p* = 0.005~0.027) of serum neutralizing activity compared with the negative control sera (Figure 1C).

For the Omicron (B.1.1.529) variant, the average NT_50_ values were calculated as 103.10 ± 8.25 for BBIBP-CorV, 99.29 ± 7.27 for WIBP-CorV, and 107.70 ± 9.25 for CoronaVac, which were statistically significantly higher (around two-fold, *p* = 0.009~0.014) than that of the negative control serum samples (average NT_50_: 47.25 ± 8.69) (Figure 1D).

Collectively, compared with unvaccinated and uninfected controls, primary vaccination with either type of inactivated vaccine could induce significantly higher levels of serum neutralizing activity against wildtype SARS-CoV-2, and Delta and Omicron (B.1.1.529) variants; meanwhile, no statistically significant difference was observed among the distinct types of vaccine (Figure 1B–D).

### 3.3. Immune Evasion by the Delta and Omicron (B.1.1.529) Variants for All Three Inactivated Vaccine Types

**Figure 2:**
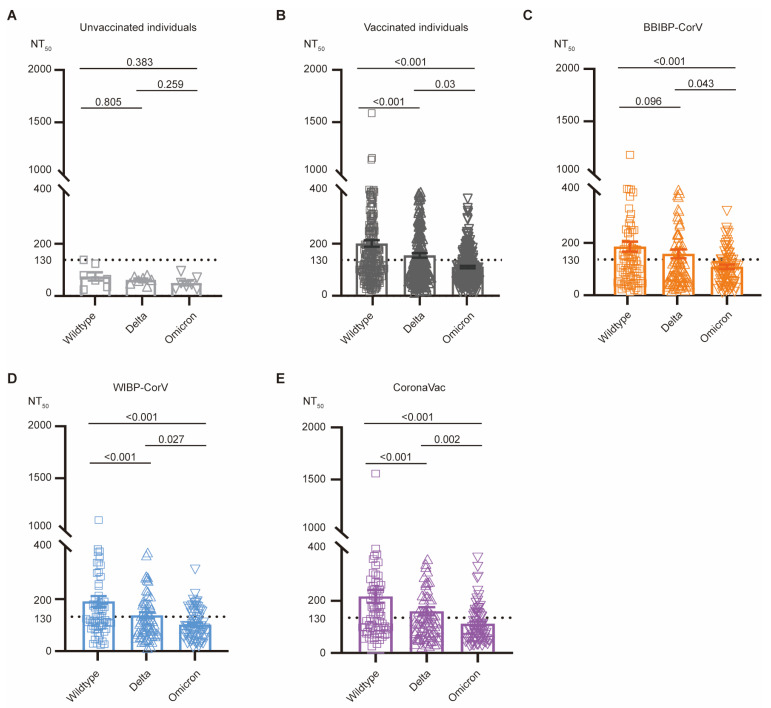
Reduced serum neutralizing activity against Delta and Omicron (B.1.1.529) variants. (**A**–**E**) Comparison of serum neutralizing titers of uninfected unvaccinated individuals (light gray, **A**), all vaccinated individuals (dark gray, **B**), BBIBP-CorV-vaccinated donors (orange, **C**), WIBP-CorV-vaccinated donors (blue, **D**), and CoronaVac-vaccinated donors (purple, **E**). NT_50_ values against wildtype SARS-CoV-2 (square), and Delta (up-pointing triangle) and Omicron (B.1.1.529) variants (down-pointing triangle) were compared using Kruskal–Wallis test.

We then compared the serum neutralizing activities against Delta and Omicron (B.1.1.529) variants with those against wildtype SARS-CoV-2. As expected, our recruited negative-control donors showed background levels of serum neutralization against wildtype SARS-CoV-2, and Delta and Omicron (B.1.1.529) variants (Figure 2A).

For all vaccinated donors that the we recruited, the serum neutralization (NT_50_) against Delta variant (average NT_50_: 147.30 ± 8.73) showed only a slight reduction, compared with those of wildtype SARS-CoV-2 (average NT_50_: 192.40 ± 12.22) (Figure 2B). This observation is consistent with the fact that the S protein of Delta has only seven amino acid substitutions and remains sensitive to most monoclonal antibodies [29]. Specifically, for BBIBP-CorV vaccinees, no significant reduction (*p* = 0.096) was observed between neutralization against wildtype SARS-CoV-2 and the Delta variant (Figure 2C). However, for the WIBP-CorV (average NT_50_: 189.90 ± 21.13 for wildtype SARS-CoV-2; 135.20 ± 12.71 for Delta) and CoronaVac vaccines (average NT_50_: 211.70 ± 24.26 for wildtype SARS-CoV-2; 154.20 ± 15.54 for Delta), around a 30% reduction (*p* < 0.001) in serum neutralization against the Delta variant was measured with statistical significance (Figure 2D,E).

The Omicron (B.1.1.529) variant has numerous mutations on its S protein, thus exhibiting striking antibody-evasion capacity [29]. As expected, regardless of the vaccine type, the serum neutralizing activities against the Omicron (B.1.1.529) variant (average NT_50_: 110.00 ± 8.47 for BBIBP-CorV; 99.29 ± 7.27 for WIBP-CorV; 107.70 ± 9.25 for CoronaVac) were significantly reduced by approximately 50% (*p* < 0.001) compared with wildtype SARS-CoV-2 (Figure 2B–E). Together, our results suggested that the serum neutralizing activities induced by all three inactivated vaccines were significantly reduced against the Delta and Omicron (B.1.1.529) variants.

### 3.4. No Significant Effect of Age, BMI, and Gender on Inactivated Vaccine-Induced Serum Neutralization

To examine the effect of vaccinees’ age, body mass index (BMI), and gender on the inactivated vaccine-induced serum neutralization, we performed a linear regression analysis and showed that, for all the three types of inactivated vaccines that we studied here, no significant correlation was observed between age/BMI and serum neutralizing titer (Figure S1).

We then performed a comparison to examine the difference in serum neutralization between males and females (Figure S2). As is shown, for these three types of inactivated vaccines, both male and female volunteers displayed similar levels of serum neutralization with no statistically significant difference. Together, in our study, age, BMI, and gender had no effect on the inactivated vaccine-indued serum neutralization.

### 3.5. Durability of Serum Neutralizing Activity After Inactivated Vaccination

Since the volunteers in this study donated their blood at different time points after primary vaccination, we further investigated the long-term immunity induced by the inactivated vaccines, and grouped volunteers into three different time periods: 0~100 days, 100~150 days, and >150 days after 2nd-dose inactivated vaccination (Figure S3).

**Figure 3:**
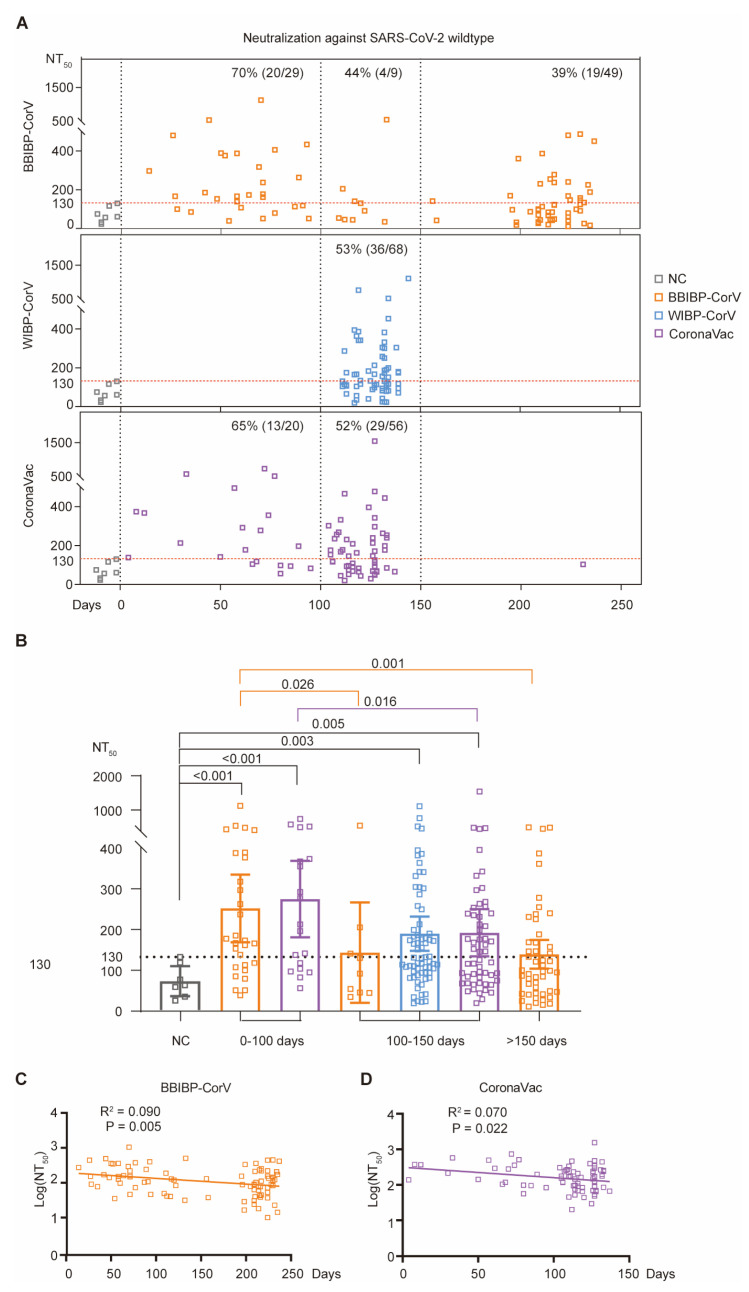
Durability of serum neutralizing activity against wildtype SARS-CoV-2. (**A**) Serum neutralization against wildtype SARS-CoV-2 at different time points post-vaccination. Each dot represents a donor: uninfected unvaccinated NC donors (gray), and BBIBP-CorV- (orange), WIBP-CorV- (blue), and CoronaVac-vaccinated donors (purple). For each donor, *x*-axis represents the number of days after primary vaccination for blood donation; meanwhile, the *y*-axis shows the measured serum neutralizing titer (NT_50_ value) against wildtype SARS-CoV-2 (square). Three different time periods are separated by vertical dashed lines: 0~100 days, 100~150 days, and >150 days after 2nd-dose inactivated vaccination. The NT_50_ values of all NC serum samples (gray square) are below 130 (horizontal dashed line) and the percentages of the donors with serum neutralizing titer higher than 130 are shown within each group. (**B**) Comparison of serum neutralizing titers against wildtype SARS-CoV-2 for different groups of donors. The *p*-values are calculated using Kruskal–Wallis test. (**C**,**D**) Correlations between the days post-vaccination (*x*-axis) and the log(NT_50_ against wildtype SARS-CoV-2) of BBIBP-CorV (**C**) or CoronaVac (**D**) (*y*-axis).

For BBIBP-CorV, the serum neutralizing activities against wildtype SARS-CoV-2 during 0~100 days after primary vaccination (average NT_50_: 252.10 ± 40.50) were statistically significantly higher than those of negative controls (average NT_50_: 70.62 ± 13.49) (Figure 3A,B). However, the serum neutralization for the group of 100~150 days (average NT_50_: 143.50 ± 53.41) and group of >150 days (average NT_50_: 139.40 ± 17.50) showed no significant difference compared with the negative control group; however, this was significantly lower than that of the group of 0~100 days (*p* = 0.026 between groups of 0~100 days and 100~150 days; *p* = 0.001 between groups of 0~100 days and >150 days) (Figure 3A,B). Moreover, the linear regression analysis showed a significant correlation between log(NT_50_) and the number of days post 2nd-dose vaccination (R^2^ = 0.090, *p* = 0.005) (Figure 3C). Importantly, the effectiveness of vaccination in around 70% of vaccinees during 0~100 days post-vaccination waned quickly afterwards, and only a proportion of vaccinees (39%) maintained their long-term serum neutralizing activity against wildtype SARS-CoV-2 after 150 days (Figure 3A).

For WIBP-CorV, all recruited volunteers donated their blood 100~150 days after 2nd-dose inactivated vaccination, and belonged to the group of t100~150 days (Figure 3A). Compared with the negative-control donors, all WIBP-CorV vaccinees (average NT_50_: 189.90 ± 21.13, *p* = 0.005) had a significantly higher level of serum neutralization against wildtype SARS-CoV-2 (Figure 3A,B). However, these WIBP-CorV-vaccinated donors showed no statistically significant difference (*p* > 0.05) with the corresponding BBIBP-CorV/CoronaVac vaccination groups (Figure 3B).

For CoronaVac vaccinees, both the group of 0~100 days (average NT_50_: 274.80 ± 44.54, *p* < 0.001) and the group of 100~150 days (average NT_50_: 192.20 ± 28.88, *p* = 0.005) exhibited significantly higher levels of serum neutralization against wildtype SARS-CoV-2, compared with the negative controls (Figure 3A,B). Further comparison showed that, compared with the donors of the group of 0~100 days, the serum neutralizing titers were reduced by around 30% in the donors of the group of 100~150 days (*p* = 0.016). No statistically significant difference (*p* > 0.05) was observed when compared with the corresponding groups (both the group of 0~100 days and the group of 100~150 days) vaccinated by the other two types of inactivated vaccines (Figure 3B). Moreover, linear regression analysis further verified the reduction in serum neutralization in a time-dependent manner (R^2^ = 0.070, *p* = 0.002) (Figure 3D).

**Figure 4:**
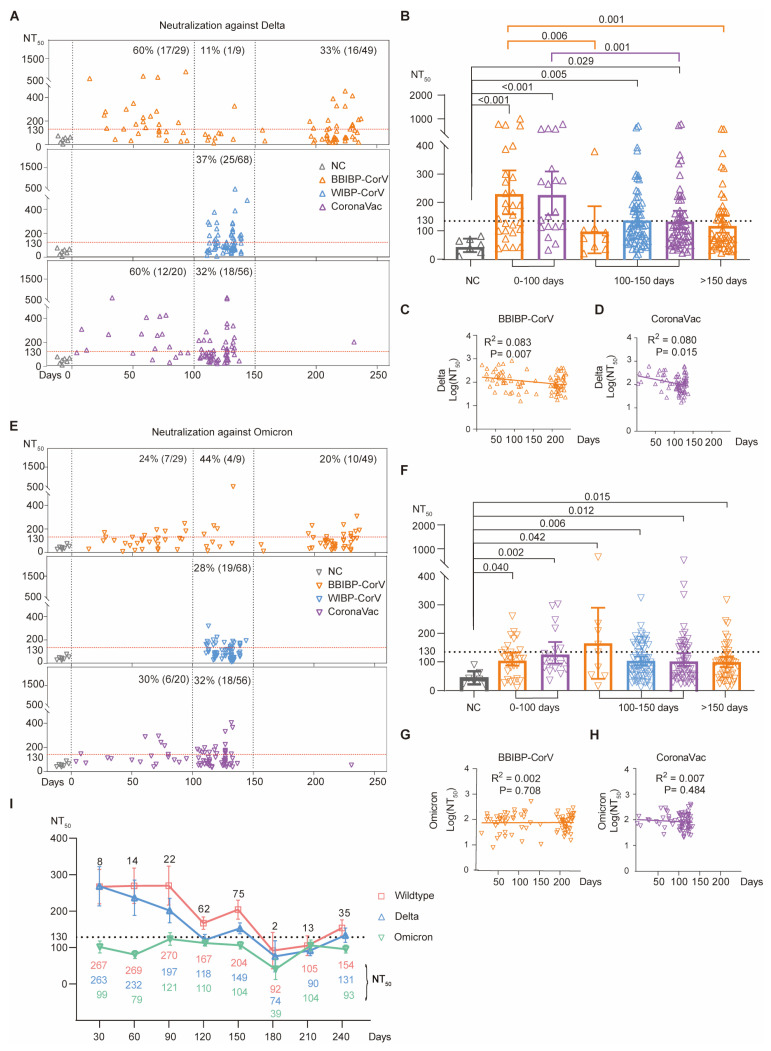
Durability of serum neutralizing activity against Delta and Omicron (B.1.1.529) variants. (**A**,**E**) Serum neutralization against Delta (**A**) or Omicron B.1.1.529 (**E**) variants at different time points post-vaccination. Each dot represents a donor: uninfected unvaccinated NC donors (gray), and BBIBP-CorV- (orange), WIBP-CorV- (blue), and CoronaVac-vaccinated donors (purple). For each donor, *x*-axis represents the number of days after primary vaccination for blood donation; while the *y*-axis shows the measured serum neutralizing titer (NT_50_ value) against the Delta (up-pointing triangle, **A**) or Omicron (B.1.1.529) (down-pointing triangle, **E**) variants. Three different time periods are separated by vertical dashed lines: 0~100 days, 100~150 days, and >150 days after 2nd-dose inactivated vaccination. The NT_50_ values of all NC serum samples (gray square) are below 130 (horizontal dashed line) and the percentages of the donors with serum neutralizing titers higher than 130 are shown within each group. (**B**,**F**) Comparison of serum neutralizing titers against Delta (**B**) or Omicron (B.1.1.529) (**F**) variants for different groups of donors. The *p*-values are calculated using Kruskal–Wallis test. (**C**,**D**) Correlations between the days post-vaccination (*x*-axis) and the log(NT_50_ against Delta) of BBIBP-CorV (**C**) or CoronaVac (**D**) (*y*-axis). (**G**,**H**) Correlations between the days post-vaccination (*x*-axis) and the log(NT_50_ against Omicron B.1.1.529) of BBIBP-CorV (**G**) or CoronaVac (**H**) (*y*-axis). (**I**) Summary of serum neutralization for all vaccinated donors at different time points. The average NT_50_ values (*y*-axis) against wildtype SARS-CoV-2, and Delta and Omicron (B.1.1.529) variants at different time points (*x*-axis) were calculated and are shown beneath the curves.

We then compared the serum neutralizing activities of distinct groups against the Delta variant (Figure 4A–D). A similar neutralization pattern as for wildtype SARS-CoV-2 was observed for the Delta variant. For BBIBP-CorV vaccinees, higher levels of serum neutralization were observed in 60% (17/29) of donors during 0~100 days, while this percentage was reduced to 33% (16/49) after 150 days (Figure 4A). Similarly, for CoronaVac vaccinees, 60% (12/20) of donors had increased levels of serum neutralization during 0~100 days, while only 32% (18/56) of donors had increased levels after 150 days (Figure 4A).

We further compared the BBIBP-CorV-induced neutralization titer (NT_50_). As is shown, BBIBP-CorV vaccination induced a statistically higher level of serum neutralization in the group of 0~100 days than the group of 100~150 days (*p* = 0.006) and the group of >150 days (*p* = 0.001, orange in Figure 4B). Similarly, CoronaVac vaccination also induced a statistically higher level of serum neutralization in the group of 0~100 days than the group of 100~150 days (*p* = 0.001, purple in Figure 4B).

Consistently, the linear regression analysis verified the decreased serum neutralization over time (R^2^ = 0.083, *p* = 0.007 for BBIBP-CorV; R^2^ = 0.079, *p* = 0.015 for CoronaVac) (Figure 4C,D).

Nevertheless, during the same period of time post-vaccination (0~100 or 100~150 or >150 days), there was no statistically significant difference in serum neutralization against the Delta variant among the three distinct types of inactivated vaccines (Figure 4B).

Finally, we compared the serum neutralizing activities against the Omicron (B.1.1.529) variant. Compared with the negative controls, each group of vaccinees showed significantly (*p* = 0.002~0.042) higher levels of Omicron neutralization (Figure 4E,F). In view of the striking antibody-evasion capacity of Omicron, the serum neutralizing titers against the Omicron variant were generally lower than those for wildtype SARS-CoV-2 and the Delta variant (Figure 2), and most vaccinees almost fully lost their serum neutralization ability against the Omicron variant (Figure 4E). However, some vaccinated donors still maintained their robust serum neutralizing activity against the Omicron variant (Figure 4E). However, since the serum neutralizing titers against the Omicron variant were very low, there was no significant correlation between the serum neutralization titer and vaccination duration (R^2^ = 0.002, *p* = 0.708 for BBIBP-CorV; R^2^ = 0.007, *p* = 0.484 for CoronaVac) (Figure 4G,H).

Taken together, these results suggested that the serum neutralization of all three tested inactivated vaccines, namely BBIBP-CorV, WIBP-CorV, and CoronaVac, waned quickly as time elapsed (Figure 4I), and no significant difference was found among the three different types of inactivated vaccines.

### 3.6. Newly Emerged Omicron Variants Fully Evaded the Serum Neutralization

**Figure 5:**
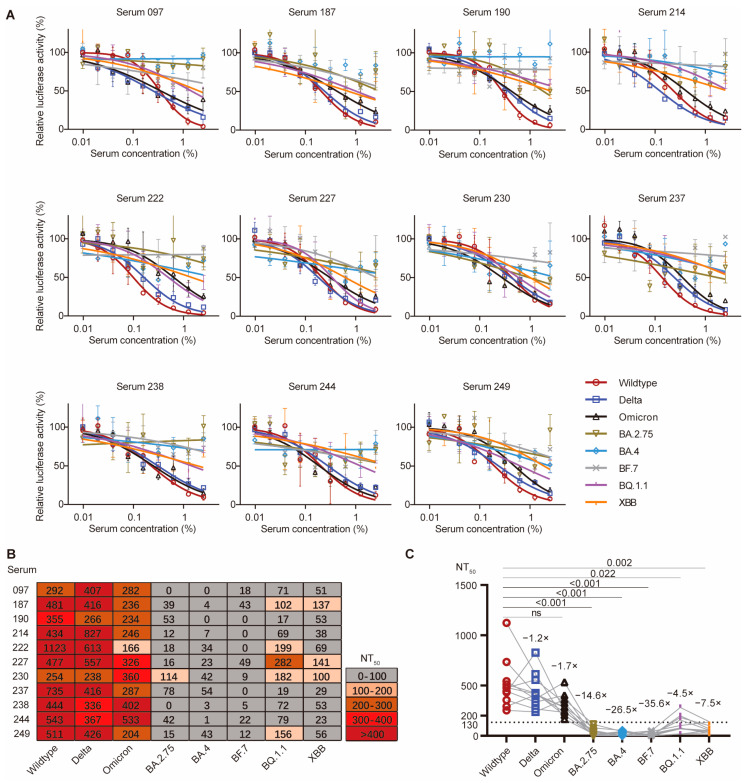
Serum neutralization against several newly emerged Omicron variants. (**A**) Pseudovirus-dependent in vitro neutralization assays against wildtype SARS-CoV-2, Delta, Omicron (B.1.1.529), BA.2.75, BA.4, BF.7, BQ.1.1, XBB pseudoviruses in the presence of different serum dilutions of the selected top 11 neutralizers. The highest serum concentration (*x*-axis) was 2.5% with a 1:2 serial dilution for eight dilutions in total. The neutralization data were analyzed based on the relative luciferase activity (*y*-axis) using Prism software by nonlinear regression (curve fit) method. (**B**) A heat map for serum NT_50_ values of the selected top 11 neutralizers. Each row represents a serum sample, while each column represents neutralization assays against an indicated SARS-CoV-2 variant. Based on the performed pseudovirus-dependent in vitro neutralization assays in (**A**), the serum neutralization titer NT_50_ value for each serum sample against a certain SARS-CoV-2 variant was calculated using Prism software. Higher NT_50_ value means more potent serum neutralizing activity. The serum exhibiting no neutralization activity has NT_50_ lower than 100. (**C**) Comparison of the calculated NT_50_ values between wildtype SARS-CoV-2 and the other different variants. The *p*-values were calculated using the Friedman test and Dunn’s multiple comparisons test, and no significant difference (n.s.) among the NT_50_ values for wildtype SARS-CoV-2, and Delta and Omicron variants was observed.

We then examined the serum neutralizing activity induced by primary inactivated vaccination against the newly emerged Omicron subvariants. To do so, we picked up 11 top neutralizers, whose serum samples exhibited robust serum neutralizing activity against wildtype SARS-CoV-2, and the Delta and Omicron (B.1.1.529) variants, and performed pseudovirus-based in vitro neutralization assays against five newly emerged Omicron variants, namely BA.2.75, BA.4, BF.7, BQ.1.1, and XBB (Figure 5A). Among the 11 selected top neutralizers (NT_50_ ranging from approximately 166 to 1123 against wildtype SARS-CoV-2, and the Delta and Omicron B.1.1.529 variants), most of them almost fully lost their serum neutralization effect against the five tested Omicron subvariants, with their corresponding NT_50_ values being reduced below 100 (Figure 5B). However, a few of them partially retained their neutralizing activity against BQ.1.1 or XBB (Figure 5B). For example, serum 227 and 230 still exhibited neutralization against BQ.1.1 and XBB, with their NT_50_ values being higher than 100 (Figure 5B). Comparison of the calculated NT_50_ of these serum samples showed no significant difference among the NT_50_ values for wildtype SARS-CoV-2, and the Delta and Omicron (B.1.1.529) variants, but a drastic and significant reduction in the NT_50_ values against Omicron subvariants (Figure 5C). Therefore, the serum neutralization induced by all three different types of inactivated vaccines were almost fully evaded by the newly emerged Omicron subvariants.

### 3.7. Limitation of This Study

This study recruited donors who has received primary inactivated vaccination. To compare the durability of three distinct types of inactivated vaccines, the donors were categorized into three groups: those at 0~100 days, 100~150 days, and >150 days after 2nd-dose inactivated vaccination. In this study, we failed to recruit donors for the group of 0~100 days post-2nd dose of WIBP-CorV vaccination and for the group of >150 days post-2nd dose of WIBP-CorV/CoronaVac vaccination. We only focused on analyzing serum neutralization in this study, but did not compare the cellular immune response across the three different types of inactivated vaccines.

Moreover, the small sample size of this study might reduce the statistical power of several comparisons in this study, thus limiting the ability to detect the significant effects of variables, such as age and BMI.

Lastly, although the assessment of serological anti-nucleocapsid antibody levels would provide more comprehensive immunologic data, we did not do so due to the limited volume of some blood samples.

## 4. Conclusions

All three distinct types of inactivated vaccines manufactured and used in China, namely BBIBP-CorV, WIBP-CorV, and CoronaVac, induced significantly increased levels of serum neutralization against wildtype SARS-CoV-2, the same strain used by all three inactivated vaccines. Within 100 days post-vaccination, the increased level of serum neutralization was observed in around 70% of vaccinated individuals. However, the serum neutralizing activity quickly waned as time elapsed, and significantly diminished against Delta and especially Omicron and its subvariants. Together, based on all the analyses, no significant difference was observed among these three distinct types of inactivated vaccines.

## Data Availability

Data will be available on request.

## Supplementary Materials

The following supporting information can be downloaded at: https://www.mdpi.com/article/10.3390/vaccines12111204/s1, Figure S1: The impact of age and BMI on the serum neutralization induced by primary inactivated vaccines; Figure S2: The impact of gender on induced serum neutralization; Figure S3: Summary of vaccinated donors.
