# Supplementary material for: Re-Evaluation and Retrospective Comparison of Serum Neutralization Induced by Three Different Types of Inactivated SARS-CoV-2 Vaccines
Source: Vaccines (Basel). 2024 Oct 24;12(11):1204. doi: 10.3390/vaccines12111204 (PMC11598389; doi:10.3390/vaccines12111204)
Supplement: Supplementary file 1 [file vaccines-12-01204-s001.zip › vaccines-3222532-supplementary.pdf]

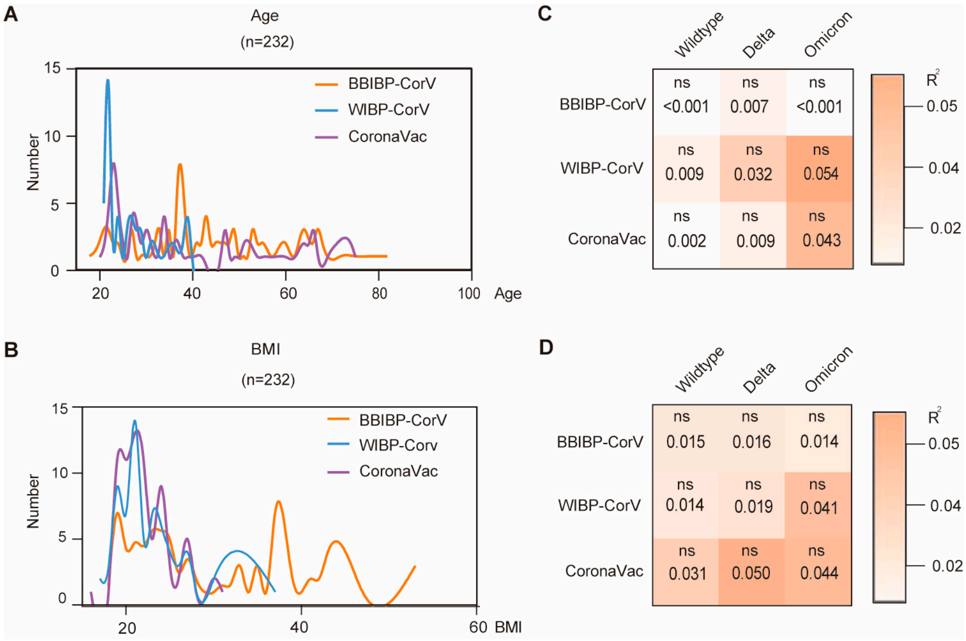

Figure S1. The impact of age and BMI on the serum neutralization induced by primary inactivated vaccines.

(A and B) Age (A) and BMI (B) distribution based on the vaccine type of donors. Orange, BBIBP-CorV; blue, WIBP-CorV; purple, CoronaVac.

(C and D) Linear regression analysis between log(NT_{50}) and age/BMI for distinct types of inactivated vaccines. The calculated R^2 are shown in a heatmap, but there is no significant correlation (n.s.).

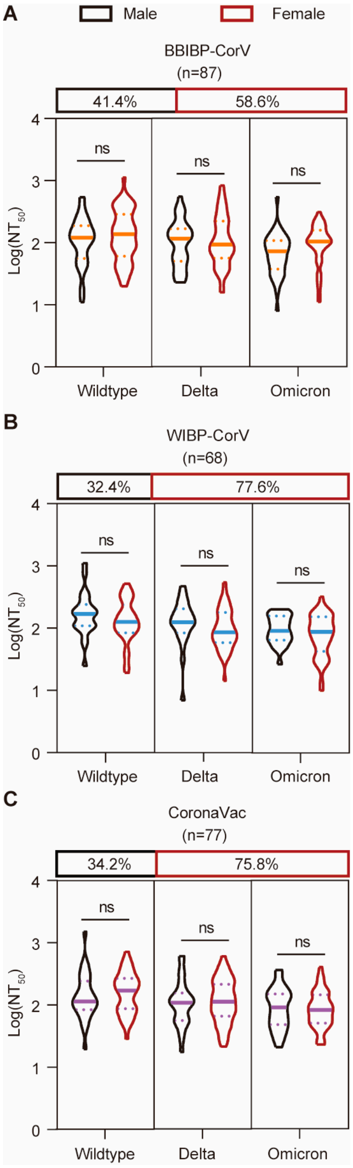

Figure S2. The impact of gender on induced serum neutralization.

Comparison of the serum neutralization effect in male and female who have received primary inactivated vaccines, BBIBP-CorV (A), WIBP-CorV (B) and CoronaVac (C). For each type of inactivated vaccine, the percentages of vaccinated male and female are shown above the violin columns. Student's t tests were performed to determine the differences of serum neutralizing titers between male and female. ns, not significant.

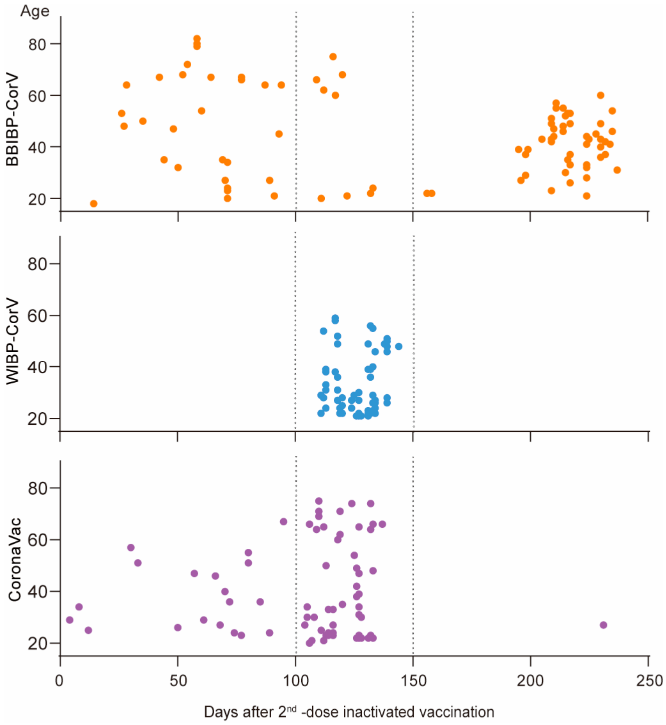

Figure S3. Summary of vaccinated donors.

All recruited vaccinated donors have received primary inactivated vaccines, BBIBP-CorV (orange) or WIBP-CorV (blue) or CoronaVac (purple). Each dot represents a donor. For each donor, *x*-axis represents the number of days after primary vaccination for blood donation; while the *y*-axis shows the donor's age. Three different time periods are separated by vertical dashed lines: 0~100 days, 100~150 days, and >150 days after 2nd-dose inactivated vaccination.
